# The related factors of new HIV infection among older men in Sichuan, China: a case–control study

**DOI:** 10.1017/S0950268822001352

**Published:** 2022-08-15

**Authors:** Feng-shun Yuan, Li Liu, Ling Su, Ya-li Zeng, Shu Liang

**Affiliations:** Sichuan Provincial Center for Disease Control and Prevention, Center for AIDS/STD Control and Prevention, Chengdu, Sichuan, China

**Keywords:** Case–control, HIV/AIDS, new infection, older man

## Abstract

Human immunodeficiency virus (HIV) has been widely prevalent among older men (aged ≥50 years old) in Sichuan Province. The study aimed to discover associated factors with the new HIV infection in older men, and provide a scientific basis for the prevention and control of acquired immunodeficiency syndrome (AIDS) in this group. A cross-sectional survey study of newly reported HIV/AIDS and general male residents aged 50 years and older was conducted between April and June 2019, with a resample of respondents to identify cases and controls, followed by a case–control study. Logistic regression was applied to analyse the association between the selected factors and new HIV infection among older men. At last, 242 cases and 968 controls were included. The results of multiple logistic regression suggested that many factors including living alone/concentrated (OR 1.56, 95% CI 1.20–2.04, *P* = 0.001), have a history of migrant worker (OR 2.10, 95% CI 1.61–2.73, *P* < 0.001), have commercial sexual behaviour (OR 1.71, 95% CI 1.32–2.22, *P* < 0.001), married (OR 0.48, 95% CI 0.37–0.64, *P* < 0.001), have a history of HIV antibody testing (OR 0.73, 95% CI 0.56–0.96, *P* = 0.026), HIV-related knowledge (OR 0.55, 95% CI 0.42–0.72, *P* < 0.001) were associated with new HIV infection among older men. The present study revealed some potential risky/protective factors altogether. The results highlighted the direction of HIV/AIDS prevention and control among older men, and it is a social issue that requires the joint participation of the whole society.

## Background

Since the emergence of human immunodeficiency virus (HIV), the causative pathogen of acquired immunodeficiency syndrome (AIDS), at the beginning of the 1980s [1, 2], with the process of globalisation, intensification of population mobility, frequent international exchanges and other factors, especially drug abuse, the epidemic has spread extensively in all regions of the world and caused serious public health and social problems. HIV/AIDS directly cost 57.6 million disability-adjusted life years in 2016 [3], and approximately 37.7 million people living with HIV and 1.5 million people were newly infected with HIV worldwide in 2020 [4]. In China, estimations indicated that the incidence of HIV was 6.422/100 000 in 2016, and people living with HIV were more than 1 million by the end of 2018 [5, 6]. In the past decade, the age characteristics of the AIDS epidemic have changed significantly, with a large number of infected people aged 50 and above [7, 8]. According to the data of UNAIDS, nearly 10% of people living with HIV worldwide were 50 years old and above [9]. In the USA, 1 in 6 HIV diagnoses were older adults, and around half of the people living with HIV were above 50 years old [10–12]. The number of HIV-infected older adults has also grown in Europe [13]. Epidemiological studies in China found an increasing trend in the number and proportion of HIV infections among older adults [5, 14]. The meta-analysis of the prevalence of HIV infection and its moderating factors among older adults in China revealed that the pooled prevalence of HIV infection in older Chinese adults was 2.1%, which was significantly higher than the general population (0.05%) [15, 16].

Sichuan Province is located in southwestern China and has the largest number of HIV-infected people in China, with more than 160 000 HIV/AIDS cases at the end of 2019 [17]. Similar to most parts of China, newly reported HIV-infected persons aged 50 years and over increased significantly, and the number increased more than 69-fold from 320 in 2008 to 22 189 in 2019, and the proportion rose from 4.1% to 59.2% [17]. The large number and high proportion of HIV infections among older adults pose considerable challenges to public health in Sichuan. Compared to older adults without HIV or younger HIV-infected adults, the HIV-infected older adults are at increased risk of adverse comorbidities, including physical and psychiatric, such as pneumonia, depression, insomnia and cognitive, psychosocial and functional decline [10, 18–22]. The meta-analysis from Wang and colleagues found that the prevalence of HIV infection among older adults in the western region of China, where Sichuan place, was the highest, reached 6.0%(95% CI 4.2–7.9), the middle region and east region were 0.5%, 1.0% respectively [15]. Faced with such a severe AIDS epidemic among older adults, Sichuan AIDS prevention and control policymakers and professionals need to take immediate action to curb the further spread of the epidemic.

However, in Sichuan, the factors associated with new HIV infection among older adults are not clear, and related studies are insufficient. To better help policymakers identify and address their specific needs for HIV prevention among older adults, it is particularly urgent to carry out studies on the related factors of new HIV infection in older adults. In addition, between 2008 and 2019, 73.0% of newly reported HIV infections among older adults in Sichuan Province were males [17], and the HIV-infected males may lead to HIV transmission between spouses. Therefore, we conducted the present case–control study among older men, to research the association between potential factors and new HIV infection.

## Methods

### Setting

During April and June 2019, we conducted a cross-sectional survey aimed at the newly reported HIV/AIDS cases between January 2018 and June 2019 and elderly male residents aged 50 years and older in Sichuan Province, China.

In the cross-sectional survey, HIV/AIDS cases were newly diagnosed and reported in the National Case Reporting System (NCRS) from January 2018 to June 2019; ordinary elderly male residents, were randomly selected in the HIV/AIDS cases investigation areas, who have lived for more than half a year. We excluded those (both cases and ordinary residents) who were incapacitated, severely mentally impaired, and refused to take part in the survey. In addition, we also tested ordinary residents for HIV antibody.

The survey areas were determined by a step-by-step sampling method. According to the number of HIV/AIDS cases and the proportion of older men in all cities (prefectures) of Sichuan Province, Chengdu, Zigong, Yibin, Meishan and Guangyuan were finally determined to carry out the investigation. Then three counties or urban districts were selected in each city by the same method. Finally, according to the number of HIV/AIDS cases, geographical location and traffic conditions in each township and community of the 15 counties (districts), two townships/communities were selected in each county (district). Fifteen counties (districts) for HIV/AIDS cases investigation, 30 townships/communities for general older men investigation, a survey of 100 older men was conducted at each township/community by convenience sampling, and a total of 3000 older men were interviewed.

The limiting antigen avidity enzyme immunoassay (LAg-Avidity EIA) was developed from the HIV-1 subtypes B, E and D IgG-capture enzyme immunoassay by the US Centers for Diseases Control and Prevention [23]. In 2020, LAg-Avidity EIA was used to detect new infections in newly reported HIV/AIDS cases in the odd months of 2018–2020, and the reagent was US Maxim HIV-1 LAg-Avidity EIA kits. The blood sample used in LAg-Avidity EIA was from HIV-positive cases retained in the −20 °C refrigerator carefully by the AIDS confirmatory testing laboratories in the Whole of Sichuan Province. The new infection could be determined when experimental results showed that normalised optical density was less than 1.5, and the infection time was within 130 days [24].

### Data collection

An interview-administered questionnaire was applied to obtain the required data. A pre-investigation was conducted before the formal investigation, and the questionnaire was evaluated and revised according to the results. The interview was completed by public health personnel who have been trained uniformly by the research group. The investigators came from the county-level Center for Diseases Control and Prevention (CDC) and primary medical and health institutions (township health centres or community health services) in the investigation areas.

Except for the questions on HIV infection, the questionnaire for HIV/AIDS cases was the same as general residents, including socio-demographic characteristics (age, gender, occupation, education and marital status), daily activities, living status, history of migrant worker (being away from home for six months or more), HIV-related knowledge and attitudes, and income. Commercial sexual behaviour and HIV antibody testing (excluding the testing to be diagnosed) in the past six months were also inquired in the questionnaire. The awareness of HIV-related knowledge was judged by the eight questions in the ‘AIDS Monitoring and Evaluation Framework of China (trial use)’, and those who correctly answered six or more questions were considered to be aware of HIV-related knowledge [25].

The interview was completed face-to-face by investigators in a private environment. The questionnaire did not contain any personal information, such as the name, ID number, current address or telephone number, to fully protect the privacy of the respondents.

### Selection of cases and controls

Cases and controls were identified by resampling the investigated HIV/AIDS cases and general older men. Cases were selected from older adult HIV/AIDS cases newly reported to the NCRS and determined to be newly infected by LAg-Avidity EIA, and 242 cases were selected finally. Controls were selected among the 3000 general older men (HIV-positive ones were excluded) by age (±1 year) in a ratio of 1:4, and the number of control was 968.

### Statistical analysis

The Chi-square test was applied to measure the unadjusted associations between the new HIV infection of older men and categorical variables. The Cochran-Armitage test was used to identify the potential change in the trend of the proportion of commercial sexual behaviour and HIV-related knowledge among ordinal categorical variables. The adjusted associations for the study variables and new HIV infection of older men were measured by Multiple logistic regression. The backward variable selection strategy was employed to include variables in the regression model. Odds ratio (OR) and 95% confidence interval (CI) were calculated. *P* < 0.05 was considered statistically significant. All analyses were conducted using SPSS 23.0 (SPSS IBM Inc., Armonk, New York).

## Results

There were 242 cases and 968 age-matched controls in the analysis. Overall, most cases (72.2%) and controls (63.9%) came from rural area (*P* = 0.016), 158 cases (65.3%) and 583 controls (60.2%) were illiterate or had elementary education (*P* = 0.003), most cases (62.0%) and controls (79.1%) were married with spouse (*P* < 0.001), 163 cases (67.4%) and 581 controls (60.0%) on HIV-related knowledge (*P* = 0.036), 172 cases (71.4%) and 75 controls (7.8%) had commercial sexual behaviours in the past six months (*P* < 0.001). Trend analysis showed that with the increase in age, the proportion of HIV-related knowledge awareness in both cases (*Z* = −4.67, *P* < 0.001) and controls (*Z* = −2.05, *P* = 0.020) showed a downward trend, while the changing trend of the proportion of commercial sexual behaviours was not statistically significant (all *P* > 0.05).

### Result of univariate analysis

Table 1 illustrates the unadjusted associations of demographic, behaviour and attitude-related variables with new HIV infection in older men. Accordingly, significant (unadjusted) associations are found between the new HIV infection and several study variables, including place of residency, occupation, marriage status, living status, history of the migrant worker, frequency of leisure activities, attitude to finding a lover/female sex worker (FSW), commercial/non-commercial sexual behaviour, HIV antibody testing, HIV-related knowledge and the number of ways to acquire it (*P* < 0.05 for all).

**Table 1.** Unadjusted association between potential variables and new HIV infection of older men

Variables	Cases (%)	Controls (%)	*χ*^2^ value	*P*-value
Age group			0.33	0.997
50–54	38 (15.7)	151 (15.6)		
55–59	40 (16.5)	160 (16.5)		
60–64	35 (14.5)	145 (15.0)		
65–69	58 (24.0)	236 (24.4)		
70–74	37 (15.3)	135 (13.9)		
≥75	34 (14.0)	141 (14.0)		
Place of residency			5.76	0.016
Urban	66 (27.8)	345 (36.1)		
Rural	171 (72.2)	610 (63.9)		
Occupation			13.90	<0.001
Farmer	171 (70.7)	557 (57.7)		
Other	71 (29.3)	411 (42.3)		
Education			2.09	0.148
Illiterate and primary school	158 (65.3)	583 (60.2)		
Junior high school and above	84 (34.7)	385 (39.8)		
Marriage status			30.75	<0.001
Single/divorced/widowed	92 (38.0)	202 (20.9)		
Married with spouse	150 (62.0)	764 (79.1)		
Living status			55.09	<0.001
Live with family/spouse	165 (68.5)	845 (88.0)		
Live alone/concentrated	76 (31.5)	115 (12.0)		
History of migrant worker			26.76	<0.001
No	174 (71.9)	831 (85.8)		
Yes	68 (28.1)	137 (14.2)		
Frequency of leisure activities			15.07	<0.001
Every day	43 (17.9)	293 (30.5)		
Occasion/none	197 (82.1)	665 (69.5)		
Income (Yuan/month)			2.23	0.328
<1000	110 (45.8)	386 (41.0)		
1000–2999	69 (28.8)	278 (29.5)		
≥3000	61 (25.4)	278 (29.5)		
Attitude to finding FSW			125.14	<0.001
Acceptance	105 (43.4)	118 (12.2)		
Neutral/opposition	137 (56.6)	850 (87.8)		
Attitude to finding a lover			58.49	<0.001
Acceptance	66 (27.3)	87 (9.0)		
Neutral/opposition	176 (72.7)	881 (91.0)		
Commercial sexual behaviour			474.79	<0.001
No	69 (28.6)	882 (92.2)		
Yes	172 (71.4)	75 (7.8)		
Non-commercial sexual behaviour			34.47	<0.001
No	213 (88.4)	938 (97.2)		
Yes	28 (11.6)	27 (2.8)		
HIV antibody testing			93.33	<0.001
No	231 (95.9)	618 (64.1)		
Yes	10 (4.1)	346 (35.9)		
HIV-related knowledge			4.40	0.036
Unknown	79 (32.6)	387 (40.0)		
Known	163 (67.4)	581 (60.0)		
Number of ways to acquire HIV-related knowledge			35.76	<0.001
Less than two	132 (54.8)	325 (33.9)		
Two or more	109 (45.2)	635 (66.1)		

*Note*: Part of the data is missing, and the actual valid data was used for analysis.

### Result of multivariate analysis

Table 2 illustrates the adjusted associations between the study variables and new HIV infection in older men. Most noticeably, live alone/concentrated (OR 1.56, 95% CI 1.20–2.04, *P* = 0.001), have a history of migrant work (OR 2.10, 95% CI 1.61–2.73, *P* < 0.001), have commercial sexual behaviour (OR 1.71, 95% CI 1.32–2.22, *P* < 0.001) were significantly associated with a higher risk of new HIV infection among older men. Furthermore, married (OR 0.48, 95% CI 0.37–0.64, *P* < 0.001), have a neutral/opposition attitude to finding FSW (OR 0.36, 95% CI 0.27–0.48, *P* < 0.001), have a history of HIV antibody testing (OR 0.73, 95% CI 0.56–0.96, *P* = 0.026), HIV-related knowledge (OR 0.55, 95% CI 0.42–0.72, *P* < 0.001), and have two or more ways to acquire HIV-related knowledge (OR 0.42, 95% CI 0.27–0.66, *P* < 0.001) were found to be inversely associated with the new HIV infection in older men.

**Table 2.** Adjusted association between the study variables and new HIV infection of older men

Variables^a^	OR^b^	95% CI^c^	*P*-value
Marriage status			
Single	1	–	–
Married	0.48	0.37–0.64	<0.001
Living status			
Live with family/spouse	1	–	–
Live alone/concentrated	1.56	1.20–2.04	0.001
History of migrant worker			
No	1	–	–
Yes	2.10	1.61–2.73	<0.001
Attitude towards finding FSW			
Acceptance	1	–	–
Neutral/opposition	0.36	0.27–0.48	<0.001
Commercial sexual behaviour			
No	1	–	–
Yes	1.71	1.32–2.22	<0.001
HIV antibody testing			
No	1	–	–
Yes	0.73	0.56–0.96	0.026
HIV-related knowledge			
Unknown	1	–	–
Known	0.55	0.42–0.72	<0.001
Number of ways to acquire HIV-related knowledge			
Less than two	1	–	–
Two or more	0.72	0.55–0.74	0.015

a The full model included marriage status, living status, history of migrant worker, attitude towards finding FSW, commercial sexual behaviour, HIV antibody testing, HIV-related knowledge and the number of ways to acquire it.

b OR odds ratio.

c CI confidence interval.

## Discussion

The results of the current study suggested that several variables including, HIV-related knowledge and the number of ways to acquire HIV-related knowledge, marriage status, living status, history of migrant worker, attitude to finding FSW, commercial sexual behaviour, and HIV antibody testing may affect the risk of new HIV infection among older men.

This study revealed that HIV-related knowledge was inversely associated with new HIV infection in older men. Published studies showed that HIV-related knowledge and the awareness of the risk of HIV infection among older adults were generally low [26]. They believed that AIDS was a disease of the young people, placing themselves at risk of HIV unknowingly [27–29]. What's more, older adults were typically omitted from HIV prevention programs [30]. Most studies focused on the awareness rate of HIV-related knowledge among different populations and its influencing factors, and rare concern on the specific impact of HIV-related knowledge on new HIV infection in older men. Therefore, the present case–control study was conducted on new HIV infections within 130 days and ordinary residents. The results showed the awareness rate of the new infection (67%) was higher than that of the control group (60%). It may be that at the time of investigation, the cases had already known their infection status, took the initiative to learn about HIV-related knowledge, and may even have received follow-up services from medical staff. However, the multivariate logistic regression analysis, controlling for other influencing factors, showed that HIV-related knowledge was still a protective factor for HIV infection in older men. The study also found that the number of ways to acquire HIV-related knowledge has a protective effect on new HIV infection in older men, indicating that various ways for AIDS publicity and education were effective. Due to low educational levels and poor knowledge understanding ability, the older adults in rural areas of Sichuan Province often had few ways to acquire HIV-related knowledge, which seriously affected their acquisition of AIDS knowledge. It suggested that public health personnel could integrate HIV-related knowledge into various forms when carrying out AIDS publicity and education, spread it to the elderly through different channels, and continuously improve their awareness of HIV prevention.

Heterosexual transmission was a dominant way of HIV transmission among older men, and many studies have indicated the paramount transmission mode was commercial heterosexual contact [15, 17, 31, 32]. It is estimated that there are 4 million to 10 million FSWs in China, and the HIV infection rate is six times higher than that of the general population, which was an important influencing factor for the infection of clients [33]. An important result of the current study was the association between commercial sexual activity and the risk of new HIV infection in older men. On the one hand, older men still have sexual needs [34, 35], which could not be satisfied due to single, widowhood or spouse's physiological reasons. The behaviour of finding FSW was easy to occur under the influence of the external environment, and the present study found single (unmarried, divorced, widowed) was a risk factor for new HIV infection in older men. On the other hand, most older men have a stable financial situation. FSWs could be easily met in small towns and villages at an affordable price, leading to older men being inclined to have unsafe sexual practices with FSWs [36]. Older men have a high rate of exposure to sex workers with a low rate of condom use [37], resulting in an increased risk of HIV infection. The proportion of FSWs who use drugs was high in Southwest China. The overlap of frequent commercial sexual behaviour and drug use may contribute to the rapid spread of HIV in the region. Because of drug funding, these FSWs were more likely to work in low-end places, serve more clients, and have a longer commercial working life. Both will increase the risk of HIV infection among older men who had commercial sexual behaviour [38]. At the same time, this study found that a neutral or disapproval attitude to finding FSW was a protective factor for new HIV infection in older men, which may be related to the lower possibility of commercial sexual behaviour for those who maintain a neutral/opposing attitude. It showed that the attitude to finding FSW has an important influence on the actual behaviour. It is necessary to strengthen the education of sexual morality in the prevention and control of AIDS.

After controlling for other variables, a significant association was observed between living alone/concentrated and the risk of new HIV infection. As the harbour of the soul, the family is the place of psychological sustenance. They lack the support, help, care and love from their families and relatives. Older men tend to have emptiness, monotonous life, lack of spiritual life, coupled with the influence of unhealthy behaviour outside, leading to high-risk sexual behaviour, particularly in under-developed western regions of China [39, 40]. Older men who lived alone have been diagnosed with HIV. They faced the dual pressures of lack of family support and HIV infection, which were detrimental to their physical health and quality of life [40]. Coupled with the weaker psychological endurance, it may even cause worse effects such as revenge [41]. Therefore, it is necessary to pay attention to the AIDS publicity and education work for older men living alone/concentrated and strengthen humanistic care.

The present study also showed that the history of migrant worker was a significant risk factor for new HIV infection among older men. These people are far away from home, their spouses are not around, and they are prone to commercial sex under the dual pressure of life and work. Studies have shown that population mobility promotes the spread of sexually transmitted diseases and other infectious diseases, including AIDS, and the infection rate of this population is high [33]. Male-dominated construction workers and long-distance truck drivers are more likely to be exposed to FSWs. With frequent sexual contact and lower HIV infection risk awareness, self-protection awareness and condom use rate, they generally have higher HIV infection rates [42]. It is suggested that in the prevention and control of AIDS, it is necessary to pay attention to older men with the history of migrant worker and strengthen publicity, intervention, and HIV testing for them.

In our study, the history of HIV antibody testing (in the past six months) was associated with a lower risk of new HIV infection. Sichuan has carried out an expanded screening strategy for many years. Whether they were active or passive participants in testing, it was an opportunity to receive AIDS publicity and education, which deepened their awareness of AIDS. An important purpose of AIDS publicity and education is to raise awareness of AIDS prevention. Voluntary counselling and testing (VCT) is a concrete manifestation of self-protection awareness in AIDS prevention. Currently, the detection of HIV/AIDS cases in older men in Sichuan Province is mainly passively diagnosed in general hospitals, and the proportion of VCT is relatively low [17]. It is suggested that it is necessary to strengthen the publicity and education of AIDS knowledge and related risk behaviours among older adults, create a good atmosphere for AIDS prevention and control, and improve the awareness of active HIV testing.

There are, nonetheless, some limitations that should be acknowledged. This study was conducted through a retrospective cross-sectional survey, and recall bias was unavoidable. The investigators of the cases and controls are different, the former is the staff of the county-level CDC, and the latter is the staff of the primary medical and health institutions. Despite the unified training, the ability of different investigators also has a certain impact on the investigation. The content of this survey about personal sexual behaviour is sensitive, and it is difficult to guarantee that the results obtained are real. Cases and controls were age-matched, and the effect of age on new HIV infection has not been studied. And controls were selected by convenience sampling methods that can lead to a sampling bias. In addition, no information on injection drug use was collected in the study, but the influence on the results of this study may be small, because the anti-drug work has always been under high pressure state in China, the proportion of traditional injection drug use is getting smaller and smaller, and the survey points were not serious drug epidemic area.

## Conclusions

This innovative study used new HIV-infected individuals as cases to study the influencing factors of HIV infection among older men. We revealed that the factors affecting their infection include personal behaviours, attitudes and external factors, which can guide HIV prevention and control professionals and policymakers. For older men with the above factors, a precise AIDS prevention and control strategy could be formulated, including AIDS publicity, education and testing, to detect the infected people as soon as possible, and prevent new infections. Moreover, AIDS prevention and control is a social issue that requires the participation of the whole society, to enrich the lives of older adults in their later years, and advocate healthy lifestyles and safe sexual behaviours.

## Data Availability

The datasets used and analysed during the current study are available from the corresponding author on reasonable request.
